# Supplementary material for: Are carbon emissions trading and green financial instruments synergistic? -Comprehensive quantitative research based on content analysis
Source: PLoS One. 2024 Mar 7;19(3):e0298601. doi: 10.1371/journal.pone.0298601 (PMC10919601; doi:10.1371/journal.pone.0298601)
Supplement: S1 Data — (ZIP) [file pone.0298601.s001.zip › Data/地级市Z_F工作报告.zip!/╔╜╬≈/╦╖╓▌╩╨/└┤╫╘╣1⁄2╓┌║┼í╛┬φ┐╦╩2╛▌═°í┐_2007.docx]

各位代表：

　　现在，我代表市人民政府向大会作政府工作报告，请予审议，并请各位政协委员和其他列席人员提出意见。

　　一、2006年政府工作回顾

　　2006年，市人民政府在市委的正确领导和市人大、市政协的有力支持监督下，坚持以邓小平理论和“三个代表”重要思想为指导，按照科学发展观和构建社会主义和谐社会战略思想的要求，紧紧围绕“十一五”地区生产总值和财政总收入“两个翻番”目标，团结带领全市人民，谋全局、抓布局、促开局，全市经济社会保持了平稳、安全、优质、快速、和谐发展的大好势头，实现了“十一五”良好开局，在省政府年度目标责任制考评中进入三个优秀市行列。

　　——国民经济平稳快速增长。地区生产总值达到232.4亿元，增长16.2%，增速全省第二，净增50.75亿元，超“十一五”年度平均净增目标13.08亿元；人均地区生产总值达到15370元，净增3276元，超“十一五”年度平均净增目标1098元。剔除资源价款后，财政总收入达到51.4亿元，增长46.7%；一般预算收入达到17.01亿元，增长50.4%；两项指标增幅均居全省第一，分别高出“十一五”预期增长目标30.3和36.2个百分点。而且地区生产总值和财政收入总量位次均由全省第十位前移到第九位。规模以上工业企业增加值完成119.8亿元，增长23.6%，超“十一五”预期增长目标6.6个百分点。粮食总产7.28亿公斤，增长9.09%，创历史最高纪录。全社会固定资产投资达到95.1亿元，增长32.3%，相当于“十五”时期年均投资额的近2倍。社会消费品零售总额62.87亿元，增长16.3%。居民消费价格总水平上涨1.95%，低于全省2—3%的控制目标。

　　——资源环境得到有效保护。万元地区生产总值平均耗水量下降2.86%，万元地区生产总值污染排放总量下降30.87%，煤矿采区回采率达到50.05%。市区空气质量二级以上天数达到284天，比上年增加38天。

　　——人民生活水平稳步提高。城市居民人均可支配收入达到9852元，农民人均纯收入达到3399元，分别增长12.1%和10.3%。新增脱贫人口9273人。

　　——和谐社会建设迈出坚实步伐。人口自然增长率为6.64‰；城镇失业登记率为2.6%；高中阶段毛入学率达到63.48%，比上年提高5.8个百分点；新型农村合作医疗覆盖率达到53.27%，比上年提高16.7个百分点。安全生产形势明显好转，全年没有发生重大安全生产事故。新增城镇就业岗位2.53万个，安置下岗失业人员再就业6970人。进一步完善了城市低保、农村特困群众救助、大病救助、贫困家庭子女就学资助等十二项社会救助制度，城镇养老、医疗、失业、工伤保险覆盖率分别达到92.3%、66.4%、85.7%和69%。

　　总之，经过全市人民的共同努力，市四届人大三次会议通过的“十一五”奋斗目标，除高新技术产业增加值和能耗指标完成不够理想外，其余均圆满或超额完成了年度任务!省政府十二件实事中，涉及我市的十一件也都圆满或超额完成!市政府承诺的九件实事，除学苑小区过街天桥正在建设外，其余均圆满完成!历届政府拖欠的1.2亿元工程款全部还清！

　　需要特别说明的是，我市高新技术产业增加值和能耗指标没有完成任务，主要在于我市建市晚、基础差和产业结构的过度重型化。完成这些指标，需做长期努力。我们将把这些指标在五年内作为整体考虑，向人民交总帐。

过去一年，我们主要抓了七个方面的工作：

　　1、扎实推进社会主义新农村建设，农业农村发展水平进一步提高。集中推进了105个试点村和153个整治村的新农村建设工作，取得阶段性成果。通过认真落实各项支农惠农政策，狠抓水利基础设施建设、生态畜牧基地建设、农业产业化经营和扶贫开发工作，进一步提高了农业农村经济发展水平。优质玉米、高淀粉马铃薯、特色小杂粮、延秋瓜菜四大种植基地面积占到农作物总播面积的85%。奶牛基地规模扩大，存栏数达到15.1万头，增长14.2%；出栏肥羔羊100万只，增长3.1%，成为全国重要肥羔羊生产基地之一。完成造林40.53万亩，全省领先。农产品加工龙头企业发展到155个，其中5家被列入全省农业产业化龙头企业方阵，占全省总数的15%。年交易额5000万元以上的农产品批发市场达到18个，完成交易额16.1亿元。争取“两区”开发项目19个，总投资54.8亿元，其中7个项目当年建成或基本建成。累计建设移民新村16个，246个村、18738人实现了移民搬迁。培训农村富余劳动力5.15万人，转移安排就业3.1万人。

　　2、加快经济结构调整步伐，发展后劲进一步增强。“十一五”规划的100个重点调产项目，建成投产和即将投产的22个，完成投资28.7亿元；在建的21个，总投资94亿元。二产结构进一步优化。煤炭工业通过实施“三大战役”，产业集中度进一步提高。地方煤矿由原来的205座缩减到143座，生产能力由3312万吨提高到5588万吨，加上同煤集团和平朔公司在朔煤矿，全市已形成亿吨生产能力。电力工业7个项目前期工作进展顺利。煤化工、新材料等新兴产业发展步伐加快，平鲁6万吨聚合氯化铝和3万吨高纯二氯化硅项目完成土建，平朔利用粉煤灰提取氧化铝和高硅氧化物项目完成中试。以旅游业为主的第三产业进一步壮大。应县通过旧城改造和龙首山文化园建设，旅游业发展势头强劲；右玉生态旅游业名声日盛；怀仁金沙滩生态旅游园初具规模；朔城区东榆林水库旅游区开发正在起步。

　　3、不断加强基础设施建设，经济社会发展硬环境进一步改善。2006年共铺开城市基础设施建设重点工程11项，其中6项已竣工，朔神大道、州北街全面通车，污水处理厂、北水南调一期工程和8座公厕已投入使用，垃圾处理厂完成土建和设备安装。公路建设完成投资7.45亿元，增长33%；新增公路通车里程175公里，新增高级、次高级路面200公里；完成农村公路及通达通畅工程1608公里，新增通水泥路(油路)村62个，通达率达到82%；全市所有乡镇和86%的行政村通了客运班车。

　　4、事关全市战略全局的重大工作进展顺利，经济社会又好又快发展的基础进一步夯实。一是积极开展招商引资活动。全年共签定项目176个，总投资169.7亿美元，拟引资107.6亿美元，资金全部或部分到位的项目达107个，实际到位资金43.68亿元，进入全省两个招商引资先进市行列。二是大力推动民营经济发展。出台了促进民营经济发展的优惠政策，成立了市县两级中小企业信用担保中心，市担保中心在全省同级城市中规模最大，在解决中小企业融资难问题上实现了突破。三是开工建设了市职业技术学院，今年秋季正式招生，改写了朔州没有高等教育的历史。四是与平朔公司合作，建立了高新技术研发中心，集中进行粉煤灰、高岭土、煤矸石开发利用研究，并针对全市每年煤炭洗选产生的近2000万吨煤泥的综合利用进行攻关研发，对全市发展循环经济、建立资源节约型和环境友好型社会将发挥重要作用。五是进一步完善了全市畜禽疾控防治体系，为广大农民在畜禽养殖方面的近百亿元财产提供了保障。六是针对我市十年九旱的实际，筹资1200万元，建立了全市六县区人工增雨体系，每年可增加降雨量约10亿立方米。七是积极筹建了海关办事处和公用保税仓库，开启了商品直接进出口的通关渠道和外商自由进出的闸门，增强了对境外投资的吸引力。八是与北京军区达成分三批将市区北部1.2万亩土地收归朔州的协议，第一批3050亩土地已拿回，化解了城市建设土地制约问题。九是积极整顿税收秩序，使每年流失到秦皇岛和北京的近5亿元煤炭销售税划归朔州，同时对全市房地产和服务业税收进行了认真的整顿。十是在促进市经济开发区发展上迈出较大步伐。将红旗牧场委托经济开发区管理，既使开发区发展空间得到进一步扩大，也为红旗牧场的生存发展创造了机遇，同时还引进了投资3000多万元的玻化砖生产线，国家发改委已核准其为省级经济开发区。

　　5、大力发展各项社会事业，公共服务水平进一步提高。在市区新建了市第四小学、城区七中，全市新建改建的100所农村寄宿制学校80%投入使用；高考二本以上达线人数达到3240人，比上年增加462人，创历史最好水平。疾病防控体系和重大突发公共卫生事件医疗救治体系建设任务基本完成，市中心医院、急救中心、传染病区和5个县级医院的传染病区已经完工；改建扩建了38个乡镇卫生院和100个村级卫生室，形成了覆盖全市的医疗救治体系；新型农村合作医疗试点扩大到3个县区。启动了“万村千乡”工程，建成68个农村便民超市。组织实施市级以上科技项目66项，取得科技成果13项，申请专利46件，其中发明专利18件，比上年增长2.75倍。朔州秧歌、晋北道情申报了国家级非物质文化遗产保护项目，踢鼓秧歌、朔州喜乐等8个项目申报了省级非物质文化遗产保护项目。广电网双向数字化改造进入整体平移阶段。启动了“百万农民健身工程”，群众性体育活动丰富多彩；竞技体育实现了“零”的突破，在全省十二届运动会上获得三金、五银、七铜的好成绩。人口计生工作受到省政府表彰。统计、妇女儿童、民族、外事、气象、档案、地震、史志、老龄、残疾人、国防动员、民兵预备役建设、人防、信访、消防等各项工作都取得新成绩。

　　6、坚持依法行政，社会主义民主法制和精神文明建设进一步加强。认真执行市人大及其常委会的各项决议，自觉向人大及其常委会报告工作，接受人大监督。积极加强同市政协、各民主党派、无党派人士的联系。办理人大代表建议、意见40件，政协委员提案92件，做到了件件有回音、事事有着落。高度重视基层民主政治建设，1684个村委会圆满完成换届选举。全力推进“平安朔州”、“信用朔州”建设，全面启动“五五”普法，切实加强了社会治安综合治理，治安形势进一步好转。严密防范、坚决抵制境外敌对势力渗透、插手宗教事务，确保了社会和谐稳定。广泛开展爱国主义、集体主义和以“八荣八耻”为主要内容的社会主义荣辱观教育，精神文明建设取得积极成效。

　　7、切实加强自身建设，政府执行力、公信力和凝聚力进一步提升。全面落实党风廉政建设责任制，切实加强行政监察和审计工作，查处了一批公路“三乱”、教育乱收费和其他侵害群众利益的问题，积极开展了药品集中采购和政府工程款审计工作。廉政文化建设不断向纵深推进，“三务”公开和政风行风评议工作受到上级表彰，清房、清车、治奢三项治理取得阶段性成果。进一步深化行政审批制度改革，取消审批项目365项，改变管理方式88项，精简审批环节704个。大力推进了机关行政效能建设，出台了机关建设“八项规定”和机关工作人员“十个不准”制度，建立了首办负责制、限时办结制、责任追究制等一系列提高行政效能、遏制行政不作为和乱作为的工作机制，机关作风明显改进。

　　各位代表，成绩确实来之不易。这是市委正确领导，市人大、市政协、各民主党派和人民团体鼎力支持，全市各级各部门和广大人民群众艰苦奋斗、不懈努力的结果。在此，我谨代表市人民政府，向为朔州改革开放和现代化建设作出重大贡献的全市人民致以崇高的敬意!向所有关心、支持朔州改革发展大业的同志们、朋友们，表示衷心的感谢!

　　同时我们也清醒地认识到，目前我市仍然是一个欠发达地区，并将持续一个较长的阶段。艰苦创业、奋力爬坡，加快发展、负重赶超，将是一项长期而艰巨的任务。全市经济社会发展和政府自身建设中还存在诸多不容忽视的矛盾和问题。一是又好又快发展的基础还不够牢固。资源型产业比重较大与资源利用效率较低并存，大型企业数量不多与中小企业发展不足并存，产品科技含量不高与企业自主创新能力不强并存，人才严重短缺与本土籍高校毕业生大量异地择业并存。经济结构调整和转变增长方式的任务艰巨。二是构建和谐社会的矛盾比较突出。社会事业和公共服务投入依然不足，城乡差距在继续拉大，少数人的富有掩盖着一部分人的贫困，群众就业难、上学难、看病难的问题仍然比较突出。和谐社会建设任重道远。三是政府自身建设的问题仍然不少。一些部门重审批轻服务、重部署轻落实、重处罚轻管理，部分基层站所滥用权力、吃拿卡要、执法扰民，少数工作人员素质不高、消极怠工、衙门作风严重，有的领导干部官僚习气十足、工作心浮气躁、奢侈浪费严重。政府管理创新的步伐急需加快。对于上述矛盾和问题，我们将以对党对人民高度负责的精神，采取更加有力的措施，切实加以解决，决不辜负人民的期望。

二、2007年经济社会发展的目标任务

　　今年是深入贯彻落实科学发展观、努力构建社会主义和谐社会的重要一年，也是“十一五”各项重大战略布局全面推进、重点突破的关键一年。今年，政府工作的总体要求是：高举邓小平理论和“三个代表”重要思想伟大旗帜，全面落实科学发展观，紧紧围绕“实现两个翻番、再造一个朔州”和建设“两大基地”、加快资源型城市转型的目标，牢牢抓住加快结构调整、转变经济增长方式这条主线，扎扎实实在培育优势产业、推进新农村建设、提高城镇化水平、扩大对外开放、加强资源节约和环境保护五个方面下功夫、求突破，着力提高全市综合经济实力、可持续发展能力、和谐社会水平和人民生活质量，努力实现经济社会又好又快发展，以优异成绩迎接党的十七大胜利召开。

　　今年，全市经济社会发展的主要目标是：地区生产总值达到280亿元，增长12%；全社会固定资产投资120亿元，增长26%；工业增加值完成170亿元，增长20%；社会消费品零售总额72亿元，增长14.5%；外贸进出口总额2310万美元，增长12%；财政总收入61.87亿元，同口径增长20.3%；一般预算收入22.1亿元，同口径增长29.9%；粮食总产量稳定在7亿公斤左右；城镇居民人均可支配收入11040元，增长12%；农民人均纯收入3670元，增长8%；居民消费价格总水平涨幅控制在3%以内；万元地区生产总值综合能耗下降5.6%；二氧化硫排放量控制在16.78万吨以下，化学需氧量排放控制在1.3万吨以下；城镇登记失业率控制在4%以内；人口自然增长率控制在7‰以内。在此基础上，全面完成“十一五”规划五个方面四十四项经济社会发展年度目标。

　　为实现今年各项奋斗目标，要努力抓好八个方面的工作：

　　(一)大力发展现代农业，引深社会主义新农村建设，在全面提高农业农村发展水平上下功夫

　　构筑现代农业产业体系，提高生态畜牧经济区建设水平。抓龙头建基地，提高农业产业化水平。坚持把发展龙头企业作为新农村建设和发展现代农业的主要抓手，突出抓好中大科技、雅士利、中粮应县糖厂等10家新龙头企业的投产达产，力争销售收入均达到亿元以上；继续扶持古城、康喜、雁门等11家销售收入超亿元的企业向更高档次迈进；指导培育天鹏农牧、渝源玉米、渊源药业等15家具有一定潜力的小型龙头企业向亿元目标奋斗。积极引导龙头企业与农民结成紧密利益共同体，推动种养基地建设。种植基地要在继续抓好优质玉米、马铃薯、小杂粮、延秋瓜菜四大基地的同时，建好甜菜、葫麻基地，近期内各发展到20万亩。畜牧基地重点抓好十大标准化养殖园区、一批人畜分离园区和青贮窖的建设，推动畜牧业更好更快发展，力争到年底奶牛发展到20万头，肉羊发展到265万只，使畜牧业真正成为农民增收的主渠道，打造全省一流的畜牧强市。加快生态建设步伐。完成天然林保护、退耕还林、京津风沙源治理和“三北”防护林工程造林26.9万亩；完成1032公里通道绿化、万亩荒山绿化、100个村庄绿化、万亩环城绿化、200个新农村村镇绿化、1.6万亩厂矿区绿化和城市绿化六大绿化工程。加快服务体系建设，强化对现代农业的支撑力。继续完善畜禽疾控防治体系建设，启动市动物疫病控制中心，在抓紧现有30个畜牧兽医中心站竣工的同时再建5个中心站；全面完成人工增雨体系建设；整合基层农技推广站，年内建成13个乡镇区域推广站；建设土壤肥料动态监测网络体系；建设市级农产品质量监测中心，在县区设立监测站，在主要农产品交易市场和生产基地设立监测点。加快推进农业标准化建设，扩大应县无公害蔬菜标准化示范种植，推进平鲁“红山荞麦”、应县小石口紫皮大蒜两个具有地方特色产品的原产地产品申报工作。新增200个农民专业合作经济组织；筹建市级农业科研机构。

　　加强农村基础设施建设，提高农业综合生产能力。启动六项水利工程。一是新水源工程。新建海子湾水库、太平窑二库、下庄头水库、大梁水库和东石湖水库，增加蓄水能力7600万立方米。二是灌区节水改造工程。完成裕民灌区、桑干河灌区应县片和井灌区朔南片、应县大临河片4处首都水资源可持续利用工程，完成神头泉域整治，新增节水面积9.2万亩。三是小型水利工程。新建矿水还田工程20处、雨水集蓄工程300处，扩大灌溉面积1.2万亩。四是水保治理工程。完成治理面积60万亩。五是供水能力建设工程。新增供水能力1200万立方米，满足工农业和城市居民用水需要。六是争取引黄北干线从七里河、桑干河明渠疏水。抓好农机三项示范。实施2000亩机械化保护性耕作、牧草机械化生产和农作物秸秆机械化综合加工示范项目，农机总动力发展到175万千瓦，增长7%，耕种收综合机械化水平达到42%。提高基本农田建设质量。通过复垦开发新增耕地2万亩；实施世行7亿元改碱富民工程，加快中低产田改造。

　　加快新农村建设步伐，提高统筹城乡发展能力。要创造特色，创新方法，加强薄弱环节，巩固扩大新农村建设成果，高标准完成200个试点村和重点推进村建设任务，担起全市新农村建设排头兵重任，力争通过五年时间的努力，一部分进入小城镇行列，一部分成为中心村。扎扎实实抓好200个治理村建设，集中治理脏乱差问题，务求年内村容村貌有一个大的改观。要切实搞好规划，以县为单位，着眼县域经济发展，凸显区域优势，搞好村庄功能划分，以经济建设联动、基础设施连片、社会事业共用为目标，完成县区新农村建设总体规划、部门规划和乡村规划。要把沼气推广作为新农村基础设施建设的重要任务，整村推进，带动农村改厕、改厨、改圈、改水。要把选好带头人作为新农村建设的主要措施，为农村经济发展培育一批有头脑、懂经营、会管理的领头雁。

　　(二)加快结构调整步伐，推进产业优化升级，在加快资源型城市经济转型上下功夫

　　不断壮大优势产业。牢固确立“一抓煤、二抓电、三抓新兴产业”的调产思路，着力推动传统产业不断升级、新兴产业不断壮大，构筑多元支柱产业体系。以“规模化、现代化、增值化、安全化”为方向，加快煤炭产业升级改造。坚持走规模化发展的路子，鼓励支持平朔、金海洋、同煤三大集团收购兼并地方小煤矿，进一步提高煤炭产业集中度，提高煤矿现代化、机械化水平，提高安全生产能力和经济效益，力争“十一五”末全市煤炭生产能力达到1.8亿吨，向全国同等城市最大的新型煤炭基地迈进。年内完成两座150万吨以上矿井建设，铺开6座120万吨矿井和15座90万吨矿井建设。坚持走现代化发展的路子，完成30座矿井的综采改革，力争两年内全市所有煤矿全部实现机械化开采。坚持走增值化发展的路子，不断提升洗选加工能力，提高煤炭质量和市场竞争力，使原煤入洗率达到90%以上。坚持走安全发展的路子，建设质量标准化矿井85座，地方煤矿全部完成监控系统建设，让广大矿工不再为煤炭增长付出生命和鲜血的代价！以上数量、上环保、上节能为重点，进一步发展壮大电力工业。加快推进脱硫除尘环保发电机组和空气冷却、利用风能与矸石的节能机组、循环利用的热电联供电厂建设，重点抓好右玉2×20万千瓦、平鲁2×30万千瓦、山阴2×30万千瓦发电项目，继续推进怀仁2×60万千瓦、神头一电厂关小上大、晋能2×20万千瓦热电联供等项目的前期工作，争取“十一五”末全市电力装机容量达到700万千瓦左右，使我市成为位居全国前列的新型火电基地。以抓扩张、提档次、增份额为目标，加快新兴产业发育成长。加快现代煤化工、煤机装备制造、农畜产品加工、新材料、陶瓷、医药保健品等新兴产业的发展，务求在扩张发展的速度上有一个大的提高，在产品的档次上有一个大的提升，在全市经济构成的份额上有一个大的增长。重点抓好晋能“双10万吨”铝硅合金、平朔利用粉煤灰提取氧化铝和高硅氧化物项目、怀仁县200万吨干法水泥、金海洋集团180万吨水泥熟料等项目建设。加快旅游业发展。搞好应县木塔景区和龙首山文化园区、山阴广武古军事文化旅游区、朔城区崇福寺和东榆林水库景区、右玉生态旅游区、怀仁金沙滩生态文化旅游区、平鲁黑驼山等景区的开发。

　　全力实施“三五Ｏ”项目建设工程。抓好鹏飞公司10万吨大砂轮、同星公司5000吨土霉素、泉鑫公司5万吨莫来石等50个在建项目，力争年内全部投产。确保新科公司10万吨盐碱地专用肥和20万吨甜高粱秆转化燃料乙醇、汇源沙棘高新技术产品加工等50个新项目顺利开工，力争两年内建成投产。抓好山阴金源公司6万吨合成氨联酶和6万吨甲醇、应县梨花春黄芪口服液等50个项目的前期工作，为明年经济发展奠定基础。

　　加快九大工业园区建设。继续抓好朔城区富甲工业园区、刘家口煤电工业园区、神头电力工业园区、平鲁井坪工业园区、右玉梁威工业园区、山阴北周庄工业园区、古城工业园区、怀仁云东经济开发区、新发高新技术工业区九大工业园区建设，千方百计吸引企业入园，使其真正成为产业集聚的平台。

　　抓好三大方阵企业。要以百折不挠的毅力培育自己的“航母”企业和“旗舰”集团，增强整体竞争力。“十一五”期间，我市规划建设“三大方阵”企业12个，其中：平朔煤炭工业公司、金海洋集团2010年销售收入要达到100亿元以上，为第一方阵；同煤朔州公司、晋能朔州公司、鲁能朔州公司、国怀精煤公司销售收入要达到50-100亿元，为第二方阵；神头一电厂、神头二电厂、大唐神头电厂、蒙牛朔州公司、伊利朔州公司、古城集团销售收入要达到10-50亿元，为第三方阵。政府要努力为这些企业创造快速发展的环境和条件，在资源配置、项目布局、政务环境、指导服务上给以最大的支持，帮助这些企业通过新上项目、技改扩建、联合兼并等多种渠道，快速做大做强。

　(三)加强节能降耗减排，加大科技创新力度，在转变经济增长方式、提高可持续发展能力上下功夫

　　能源消耗居高不下、污染物无害化处理率很低、科技创新能力不强，是我市经济又好又快发展的突出弱点。坚决把能耗降下来、把排污控制住、把科技创新搞上去，迫在眉睫，刻不容缓！严格执行能耗和环保标准，从源头上加大监管监测力度。新上项目必须进行能源消耗审核和环境影响评价，达不到要求的一律不准上马；在建项目必须经过节能和环保设施验收，不合格的一律不准投产；建成项目必须按照能耗和环保标准整顿改造，仍不达标的一律关闭。要严守耕地保护红线，坚持集约用地，严厉打击违法占地，逐步建立以单位面积投资强度为标准的工商业占地准入制度和限期开发的土地召回制度。要完善相关监测、统计和考核制度，实行地区和重点企业资源消耗、污染排放定期公布制度，完不成能耗节约和环保任务的县区，停止审批新的工业项目。突出抓好煤炭、化工、陶瓷、建材、建筑等重点行业的节能减排和城市大烟囱、居民燃煤灶等方面的排污治理工作。继续打好煤炭“三大战役”，坚决淘汰小煤矿。加快水泥、电解铝、铁合金、电石等行业落后生产能力改造和淘汰步伐，关闭取缔违法集煤场和单位自备锅炉，加快桑干河及其支流七里河、恢河、元子河流域地表水环境综合治理，完成晋能公司、光达电厂、汇诚电冶、神头二电厂、大唐电厂的脱硫改造。扎实推进“蓝天碧水工程”。确保垃圾处理厂年内投入运营。在全社会开展厉行节约、反对浪费的活动，让头顶同一片蓝天的全市人民共同承担建设资源节约型、环境友好型社会的重任！

　　大力发展循环经济。发展循环经济，是我市加快城市转型、实现节能降耗减排、建立资源节约型和环境友好型社会的重要途径。要坚持资源节约、综合利用、清洁生产的原则，制定全市和各重点行业发展循环经济的具体规划。要从企业层面入手，逐步开发废弃物利用加工，形成“点”上的初级循环；从发展关联企业入手，在企业与企业之间相互利用代谢物，形成“链”上的中级循环；最终通过建立社会配套体系，对公共废弃物进行综合开发、利用、转换，形成“面”上的高级循环体系。要首先从煤炭起步，先易后难，逐步推进。引导煤炭企业在整合的过程中形成循环发展的布局，通过改革采煤方法、兴办相关地面工业，提高节约资源、循环利用的能力。重点抓好平朔、神头电厂、金海洋、刘家口四个循环经济示范园区建设，积极发展煤—电—冶金，煤—电—粉煤灰—聚合氧化铝—氧化铝—高硅物质—建材，煤—电—高岭土和水泥等材料工业，煤—煤气化—煤化工—陶瓷及民用煤气四大循环产业链，使全市循环经济有一个实质性的进展。

　　大力实施“人才强市”和“科教兴市”战略。加大科技投入，今后市财政每年用于科技研发的经费不低于本级财政预算支出的1%，并保证足额到位。大力推进科技创新，加强与科研院所、大专院校的联合协作，加快科技成果引进、消化、吸收、应用步伐，启动“中日雁门关地区生态恢复和扶贫合作项目”。进一步推进科技体制改革，突出企业科技创新的主体地位，大张旗鼓地表彰奖励一批科技项目和科研先进集体、模范个人，推动我市科技创新迈上新的台阶。继续落实有关引进人才和科技进步奖励的有关规定，确立“不求所有、但求所用”和“不求常住、但求常来”的人才引进思路，创造环境，用事业吸引人才、培育人才、留住人才。从今年起，每年吸纳一部分大学本科以上毕业生充实干部队伍，改变干部队伍知识结构，提高干部队伍整体素质。

　　(四)加大基础设施建设投入，完善中心城市功能，在加快特色城镇化进程上下功夫

　　大幅度提升中心城市建设管理水平。进一步拉开城市框架，扩大城市规模。将大运公路西移4公里，年内开工，明年上半年竣工；现大运公路市区段改造为90米宽、15公里长的城市道路，并投资亿元左右，在民福街与大运路交叉路口建设一座大型立交桥和两座平板桥。将开发路向南延伸至麻家梁煤矿，总长度达到17公里，与大运公路市区段共同构成市区南北两条主干道，整个市区形成两纵九横主框架，使中心城市面积由现在的22平方公里扩大到100平方公里。加快一批重点项目建设，完善城市功能。年内铺开市体育馆、图书馆、博物馆、新闻中心、文化艺术中心、火车站候车大厅及站前广场改造、公安技侦中心、海关商务服务大厅、大运公路市区段改造开发和旧城改造“十大重点工程”，力争2008年年底以前全部投入使用，为建市20周年大庆献礼。同时抓紧完成学苑小区过街天桥、建设路两侧开发、城市北水南调二期工程和小村、北邢家河、上庄头、下庄头四个“城中村”改造工程。通过上述一系列工程建设，在市区逐步形成七大景观，即水、桥和文化艺术中心交相辉映的七里河公园景观；占地600多亩，集新闻中心、五星级宾馆、博物馆、图书馆、行政中心和大型广场为一体的市委西区景观；较高档次的火车站候车大厅及2.7万平方米站前广场的车站景观；由容纳5000余人的体育馆及体育广场两大部分组成的体育中心景观；以职业技术学院12层教学大楼为中心的学院园区景观；以旧城东、西、南三条大街为主体，发掘恢复老城城墙原貌，作坊商铺星布其间的老城公园景观；由三座紧密相连的大桥和湿地公园共同构成的大运路立交桥景观。要放开城市户口，加快农民进城步伐，并每年安排100亩土地，为进城农民建设一批小户型经济适用住房，使市区人口尽快达到30万人以上。要围绕创建省级卫生城市、环保模范城市、生态园林城市和文明城市目标，提升管理水平，改善城市环境。突出抓好以市区主要街道和重点区域净化、绿化、亮化为主要内容的“三化工程”建设，力争两年大见成效。要教育广大市民养成爱清洁、讲卫生的良好习惯，树立现代城市文明理念。要理顺城市管理体制，市区两级要投入对接、资源共享、制度接轨、管罚合拍，下大力气解决好脏、乱、差问题，改变市区南北环境反差巨大的现状。要集中建设一批公共草坪、街心花坛，继续推进机关庭院、居民小区绿化，力争年内新增城市绿化面积88万平方米，城市建成区绿化覆盖率达到41%；人均公共绿地面积达到10平方米。要在开发路、振华街、民福街、市府街、鄯阳街“一路四街”亮化上取得突破，形成以主要街道为框架、以道路两侧建筑为重点、以广场为点缀、以广告为烘托的多层次、立体化的亮化景观。

　　加快特色小城镇建设步伐。坚持城乡统筹、以人为本、重在内涵、力求特色、生态文明的原则，进一步加快以县城为重点的小城镇建设。制定和完善县域城镇体系总体规划，加快县城基础设施配套完善、旧城改造和新区建设，切实提高县城服务功能，改善县城面貌。要因地制宜，走出各具特色的发展路子。应县要继续推进旧城改造，形成古色古香特色；右玉要巩固提高五十年建设成果，建设塞外绿色明珠；怀仁要继续走好城内现代化气息与周边生态文明相结合的路子；平鲁要加大旧城改造力度，进一步改善城市面貌；山阴要抓好大运路西移工程，搞好城市总体规划和新区建设，提升城市品位。启动县区住房公积金制度，加快城镇住房建设。与新农村建设相结合，扎实推进建制镇建设。制定优惠政策，吸引社会投入，促进产业和人口集聚，使全市城镇化水平提高到42.5%。

　　大力发展交通事业。围绕“县县通高速、乡乡通二级、村村通班车”五年总目标，抓好荣城至乌海过境高速公路、大同至呼市高速公路右玉段、应繁出口公路、大忻线朔州段改造、平鲁至神头电厂运煤专线、神池至广武一级公路、怀仁吴家窑运煤专线七项重点工程；抓好400公里县乡公路改造、500公里村与村网络公路、500公里通达通畅工程、5座危桥改造工程、100个村600公里新农村道路建设等五项农村公路工程；改建干线公路5条；新增农村客运线20条、客运车辆50部，实现90%的行政村通客车。

　　(五)不断深化改革，全面扩大开放，在增强经济发展动力和活力上下功夫

　　全面深化各项改革。在今明两年内，彻底明晰资源整合后煤炭企业的产权。继续推进国有企业深层改革，加快困难企业破产重组步伐，妥善解决好以往企业改制中遗留的人员工资、养老保险、土地权属、资产变现、债务处理等问题，最大限度地化解改革中产生的矛盾。加强企业上市辅导工作，重点帮助山西金海洋洁净煤有限公司、山西古城乳业有限责任公司、山西平朔安家岭西易煤矿有限公司、怀仁国怀精煤有限公司4家企业做好上市前的准备工作。基本完成中央、省属大中型企业分离办社会的接收和改制任务。加快粮食、供销、外贸企业和农村信用社改革步伐。搞好公务员收入分配制度改革，积极稳妥地推进事业单位改革。完成有关财政管理体制改革任务。继续深化农村综合配套改革。启动集体林权制度改革试点工作。

　　努力扩大对外开放。坚定不移地实施开放带动战略，坚持依靠优良环境、优势产业、优质服务扩大招商引资，推动国内外大企业大集团来我市投资兴业，在引进资金的同时，引进先进技术、现代管理方式和人才智力。加强对签约项目的跟踪落实，积极开展招商引资成果“落实年”活动，提高签约项目的资金到位率和开工率。尽快促成海关和商检部门朔州办事处的正式成立。认真做好项目前期准备，做到资料齐全翔实、谈判预案充分、报批手续完备。精心编制《朔州投资指南》，对投资者关心的电价、地价、供水等问题有一个科学、权威、明确的阐释，增强招商引资的指导性。加强部门之间的联合协作，提高招商引资的成功率。继续积极参加各类招商引资活动，认真组织好“首届中国(太原)国际煤炭与能源新产业博览会”、山西·珠三角投资贸易洽谈会等大型招商活动。同时，在十月份召开全市招商引资总结表彰大会。

　　大力发展民营经济。积极组织银企对接，加大金融支持中小企业的力度。继续增加中小企业发展基金，鼓励成立中小企业贷款公司，进一步缓解中小企业融资难问题。引导大企业、大项目带动中小企业发展，支持中小企业围绕大项目延伸产业链。进一步优化发展环境，加强以创业孵化、创业培训、创业指导、创业扶持、法律援助为主要内容的综合服务，让所有有志者无障碍地投身创业！让全民创业的激情形成奔腾不息的浪潮在朔州大地涌动！

　　加快市经济开发区建设。充分发挥市经济开发区区位优越、交通便利、设施完善、政策宽松的优势，以生物医药、机械制造、煤化工、建材、仓储物流等产业为依托，年内铺开投资3亿元的东区工业园基础设施建设和投资2亿元的中煤非煤工业园建设；开工建设投资2.9亿元的穆寨新农村；争取投资28亿元的同煤麻家梁矿工业园开工，努力打造晋北地区一流的外商投资乐园。

(六)大力发展各项社会事业，着力提高公共服务水平，在促进经济社会协调发展上下功夫

　　优先发展事关千秋万代的教育事业。进一步加大城市教育投入，逐步将初、高中教育向市区和县城集中，每个县区培育1—2所重点高中和2—4所重点初中，力争通过五年的努力，全市有2—3所高中成为全省名校。农村教育除个别大集镇和偏远地区外，以完全小学为主，继续抓好100所寄宿制学校建设。大力推进朔州高等师范专科学校筹建工作，力争年内开工建设新校区；争取与中央军委、国家体育总局、省体育局联合在我市建设华北航空专科学校，在兴办高等教育上再迈新步伐。加快职业教育和农村远程教育发展，力争每个县区在县城新建一所职业技术学校。不折不扣执行国家“两免一补”政策，决不让一个适龄儿童因贫失学！建立多元教育救助体系，决不让一个考上大学的贫困学子失去求学成才的机会！善待农民工子女，对进城农民工子女入学实行市民待遇，决不让农民工为建设城市流汗、为培养后代流泪！

　　加快发展直接关系人民群众生命健康的卫生事业。进一步完善公共卫生和医疗服务体系，市中心医院、疾控中心和急救中心要正式运行，提高重大疾病防控水平和救治能力。继续推进新型农村合作医疗，启动朔城区、右玉县试点。加强农村三级卫生保健体系建设，县乡村医疗卫生机构实现60%以上达标。做好重大传染病防治工作，县级传染病区要全部投入使用。加强食品药品供应监管，坚决制止医药购销中收受回扣、医疗服务中开单提成、救治过程中索要红包的问题，让人民群众享受安全、有效、方便、价廉的医疗卫生服务!

　　积极发展文化体育和其他各项社会事业。大力改善文化基础设施条件，扶持发展民办文化，建立覆盖全市的公共文化服务体系。加强对文物、非物质文化遗产和民间艺术的保护。加强文化市场管理，深入开展“扫黄打非”专项斗争。加快发展新闻出版、广播电视和哲学社会科学。突出抓好广播电视数字化整体平移，力争年内实现城市居民有线电视数字化。加快农村有线电视网络建设，提高农村“村村通”质量。积极开展群众性体育活动，努力提高竞技体育水平。强化人口和计划生育工作，统筹解决人口问题。发展外事、档案、史志、气象、测绘、地震等事业。关心支持老龄、妇女、儿童、残疾人、民族、宗教等工作。

　　（七）高度关注民生问题，努力维护社会公平正义，在建设“和谐朔州”上下功夫

　　千方百计扩大就业。坚持把扩大就业作为解决民生问题的头等大事，重点做好下岗失业和体制转轨遗留的国企职工再就业工作。高度重视并认真解决零就业家庭的就业问题，保证每个家庭起码有一人正常就业。加强职业培训，提高劳动者就业技能。推进退役军人安置改革。发展和谐劳动关系，保障劳动者合法权益。要格外关心农民工，严厉打击黑恶势力拐骗、限制人身自由、强制劳动、雇佣童工、故意伤害、克扣和拖欠工资等各种侵犯农民工利益的行为。

　　努力完善社会保障体系。认真搞好“做实企业养老保险个人帐户”试点工作，稳妥推进基本养老金计发改革。完善城镇职工基本医疗、失业、工伤、生育保险制度，力求辐射到各种所有制主体和灵活就业人员。加强城乡社会救助体系建设，继续落实好各项优抚政策。建立农村最低生活保障制度，推进城乡特困群众大病医疗救助制度，让公共财政的阳光普照城乡所有困难群体。

　　高度重视安全工作。安全是民生的第一需求。生命没有高低贵贱之分。要不断强化安全生产制度、责任、追究、惩治、监管体系建设，做到警钟长鸣，常抓不懈。抓好煤矿“一通三防”，在全部合法矿井强制推行省、市、县管理部门和煤炭企业、井下工作面五级联网的瓦斯监测与自动处理系统、产量监控系统、井下人员考勤定位系统建设。加强对危险化学品、烟花爆竹、道路交通、公共场所、特种设备等重点领域的安全管理，遏制重特大事故发生。

　　切实维护社会稳定。围绕建设“平安朔州”，全面落实社会治安综合治理的各项措施，加大公共治安投入，充实公安队伍，加强对重点地区、突出问题和多发性问题的治理，严厉打击黑恶势力和经济刑事犯罪，防止重大恶性案件发生。拓宽社情民意的表达渠道，主要领导干部要带头接访、下访，带头查办重点信访案件，下功夫解决群众反映强烈的问题，避免群体上访、越级上访。

　　扎扎实实办好十六件实事。认真落实好省政府交办我市的十一件实事：一是努力缓解人民群众看病难、看病贵问题，完成60所乡镇卫生院改扩建工程；新型农村合作医疗制度覆盖到5个县区；市县区全面推行医疗救助制度；医院药品价格平均下降30%左右。二是改善边远贫困地区学校办学条件，支持55所农村寄宿制小学配置暖气。三是关心帮助农村特困群众生活，对6.18万名农村低保对象年人均补助360元，对1.24万名农村五保对象按标施保。四是企业职工养老保险参保人数达到8万人，工伤保险参保人数达到7.5万人，其中农民工参保人数达到5万人。五是改善农村生产、生活条件，发展沼气2万户，解决10万农村人口饮水安全问题，修建通村水泥（油）路500公里，具备条件的建制村全部通公路。六是改善城镇困难居民住房状况，建设安康工程住房33.5万平方米。七是对20个地质灾害村进行集中治理，让2917户、1.1万农民住上新建和安全的住房。八是培训农民5万人，转移农村富余劳动力3万人，移民搬迁2200人。九是继续实施“万村千乡市场工程”，发展连锁农家店120家。十是实施“双千双百惠农工程”，累计发放支农贷款55亿元以上，为农民提供信贷服务超过17万户，支持65个带动农民增收的龙头企业，扶持22个农民专业合作社。十一是改善生态环境，市区空气质量二级以上天数达到286天。同时，市政府还要为群众办好五件实事：一是从神头电厂引热入市，进一步解决城市居民供热问题；二是投资亿元以上，帮助农民兴建青贮窖1万个，人畜分离养殖园区100个，并进一步完善畜禽疾控防治体系和良种繁育体系；三是在朔城区、平鲁区试行65岁以上农村居民最低生活保障制度，每人每月30元；四是为在市区从业三年以上的打工者和有自办企业的投资者等解决城市户口，实行市民待遇；五是全面完成有线电视“村村通”工程。这十六件实事，件件关系民生，事事体现民意，我们一定要用真心、动真情、下真功抓好落实，使其成为密切党群干群关系的重要载体，成为惠及千家万户的民心工程。

　　（八）加强社会主义民主法制和精神文明建设，加快依法治市步伐，在推动“三个文明”同步提高上下功夫

　　积极推进社会主义民主政治建设。市政府将认真执行市人大及其常委会的各项决定决议，主动向人大及其常委会报告工作，自觉接受人大监督；密切同人民政协、各民主党派、工商联、无党派人士和各人民团体的联系，充分发挥其参政议政作用。建立健全市长与人大代表“直通车”和政协委员接待日制度，认真办理人大代表、政协委员的建议、意见和提案，拓宽与群众沟通协商的渠道。今后凡涉及全局性的重大决策，都要预先征求人大代表和政协委员的意见，特别是不同的意见，这要作为一项制度定下来。支持人民法院、检察院依法履行职责，充分发挥工青妇等群团组织参加经济、政治、社会、文化建设的积极作用。努力扩大基层民主，完善基层群众自治组织和企事业单位民主管理制度，扩大政务、村务、厂务公开范围，保障人民群众的知情权、选举权、参与权和监督权。探索建立市政府及其主要部门新闻发布制度，进一步增加政府工作的透明度。

　　加强社会主义精神文明建设。加强社会主义核心价值观体系和思想道德体系建设，深入开展荣辱观教育，倡导以爱国主义为核心的民族精神和以改革创新为核心的时代精神，建立与社会主义市场经济相适应的社会公德、职业道德、家庭美德和个人品德，努力在全社会形成共同的理想信念、道德规范和价值取向。提高全民素质，塑造健康人际关系，提倡文明高尚的生活方式。继续探索“双应一体化”国防动员建设道路，深入开展“双服务”活动，加强“双配套”建设，推动国防后备力量建设迈向新水平。

三、加强政府管理创新与自身建设

要转变职能、创新管理，致力建设一个尽职守责、科学施政的服务型政府。自觉适应市场经济发展和社会转型的要求，切实把政府职能转变到经济调节、市场监管、社会管理和公共服务上来，加快由“全能政府”向“有限政府”的转变，着力解决好政府工作越位、缺位、错位的问题，做到守本份、尽职责，全力做好自己应该做的事情。在经济调节和市场监管上，要在退出一般性竞争领域、为市场经济主体让出更大发展空间的同时，切实当好市场经济的“服务生”，竭尽全力为市场经济主体提供信息、政策及资源配置上的服务与扶持，促进市场经济主体更快发育成长；当好市场经济的“守护神”，竭尽全力为企业排除各种干扰，维护企业合法权益，为市场经济发展创造良好外围环境；当好市场经济的“裁判员”，对市场经济运行中的各种矛盾和问题进行公平公正的裁决，合理调整各种利益关系，化解各类经济社会矛盾，维护良好经济秩序，促进公平竞争。在社会管理上，要致力维护社会安全稳定，促进社会公平正义，构建良好社会公德风尚，着力开展社会救助，促进和谐社会建设。在公共服务上，大力发展科技、教育、文化等各项事业，大力推进各项基础建设，努力为全社会提供更多公共产品。要进一步深化行政审批制度改革，减少审批事项，凡是市场主体能够自己解决的、凡是通过市场竞争机制能够完成的、凡是市场中介组织能够自律管理的，政府一概不包办代替、不插手干预，把主要精力放在提供更多公共服务和创造优良环境上。要创新管理，建立健全科学的决策机制，从源头上提高服务水平。对事关经济社会发展全局的重大问题，坚持从群众中来，到群众中去，深入调查研究，广泛集中民智，充分征询社会各界的意见；对专业性、技术性较强的重大建设项目，要组织专家进行科学评估论证，慎密决策；对事关人民群众切身利益的热点问题，建立社会公示与听证制度，让群众主动参与、积极监督；对事关改革发展的难点问题，坚持实事求是、因地制宜、量力而行，尊重群众意愿，充分发挥群众的积极性、主动性、创造性。

　　要遵纪守法、规范行为，致力建设一个依法行政、公正诚信的法治政府。认真贯彻《行政许可法》和《全面推进依法行政实施纲要》，严格依法管理经济社会事务。加强政府规章建设。围绕改革发展稳定的重大问题和群众生产生活的突出问题，及时制定相关的规范性规章制度。推进综合执法体制改革。相对集中行政执法权，努力解决多头执法、执法不公、违法执法的问题。明确执法界面，减少执法层次，切实改变行政执法“上下一般粗”的现状。建立行政执法收支分离制度，解决“以罚代改”的问题。

　　要改进作风、狠抓落实，致力建设一个纪律严明、勤政务实的效能政府。按照胡锦涛总书记提出的八个方面的优良作风和我省“狠抓落实年”、“作风建设年”的要求，在全市政府系统大兴敢抓敢管、勇于负责之风，大兴调查研究、求真务实之风，大兴锐意改革、致力创新之风，大兴艰苦创业、勤俭节约之风，大兴团结协作、合力攻坚之风，真正在实干和落实上下真功、用苦功，形成同心同德、精诚合作、共谋发展的整体合力和良好氛围。要严肃政令，从严治政。加强对县区政府和所属部门的管理约束，坚决杜绝政府权力部门化和部门权力个人化、利益化，做到有令必行，有禁必止，政令畅通。建立严格的目标责任制和责任追究制，赏罚分明，有责必问，问责必严，下决心整治“当官不负责任、掌权不受约束、只吃俸禄不干实事”的问题，下决心整治“看有无背景办事、看好处多少办事、看心情好坏办事”的问题，着力解决好群众反映比较强烈的干部不作为和乱作为问题。

　　要强化监督、严格自律，致力建设一个团结拼搏、风清气正的廉洁政府。加强党风廉政建设，建立健全教育、制度、监督并重的惩治和预防腐败体系。认真贯彻中纪委《严格禁止利用职务上的便利谋取不正当利益的若干规定》，继续加强建设工程招标、经营性土地出让、产权交易、资源使用、重大项目投资、政府采购等制度建设，从源头上预防腐败现象滋生。加强廉政文化建设和廉政教育，使每个干部，特别是领导干部，都能做到知廉耻、讲廉洁，谨言慎行，警钟长鸣，并管理教育好家属、子女和身边工作人员。加强权力监督，推进政务公开。抓好领导干部经济责任审计。大力开展专项治理，深入推进治理商业贿赂工作。认真解决严重损害群众利益的突出问题，纠正部门和行业不正之风，坚决查办违法违纪案件，严惩腐败分子。

　　各位代表，形势催人奋进，任务逼人奋发。让我们在中共朔州市委的坚强领导下，紧紧依靠全市人民，抓住机遇，迎接挑战，排难奋进，为实现全市经济社会又好又快发展而努力奋斗！
